# All that glitters is not gold! Job insecurity and well-being in STEM research fellows: a latent profile analysis

**DOI:** 10.3389/fpsyg.2024.1347966

**Published:** 2024-05-30

**Authors:** Giulia Bacci, Sara Viotti, Lara Bertola, Daniela Converso, Barbara Loera

**Affiliations:** ^1^Department of Psychology, University of Turin, Turin, Italy; ^2^Department of Management and Organisation, Rennes School of Business, Rennes, France

**Keywords:** job insecurity, academia, researchers, work-related stress, work engagement, latent profile analysis (LPA)

## Abstract

Job insecurity is now one of the major stressors affecting well-being at work. In academia, researchers appear to be in the most precarious position. To explore the relationship between job insecurity and well-being at work, we analyzed a sample of research fellows belonging to STEM disciplines in Italy. Using a latent profile approach, we identified three “hidden” subgroups: “Safe & Sound”; “Safe not so Sound” and “Neither Safe or Sound.” Compared to previous studies, our results show that even within a population of STEM researchers that tends to have good levels of employability and mobility, there are subgroups of people characterized by greater job insecurity and low work commitment, who suffer from emotional exhaustion and cynicism at work level, i.e., more exposed to the risk of burnout.

## 1 Introduction

Job insecurity has emerged as a prominent stress factor in recent years, particularly among younger workers. It embodies an individual’s subjective fear of involuntary job loss in the near future (Sverke et al., 2002). This fear signifies a potential fundamental, involuntary change in one’s current employment status, revealing the delicate nature of job stability in today’s organizational landscape. The implications go beyond mere employment concerns and affect critical work-related facets such as financial stability, social relationships and personal identity.

Classified as a stressor due to its detrimental effects on work-related resources (Vander Elst et al., 2016), job insecurity stems from a discrepancy between an individual’s experienced and desired levels of security (Hartley et al., 1991; Keim et al., 2014), exacerbating its psychological impact. Studies have equated the distress caused by fear of job loss to that experienced when unemployed, particularly during adverse labor market conditions such as economic crises (Gallie et al., 2017).

This uncertainty at work potentially leads to increased turnover intentions and reduced employee loyalty (Arnold and Feldman, 1982; Davy et al., 1997). Different research suggests conflicting results - some suggesting lower self-rated performance due to job insecurity, while others argue for increased employee effort to secure their position (Brockner, 1988; Ashford et al., 1989; Armstrong-Stassen, 1993; Rosenblatt and Ruvio, 1996; Sverke and Hellgren, 2001; Sverke et al., 2002). The complexity of this relationship requires further research.

In addition, job insecurity is pervasive in academia, particularly affecting young researchers in training, due to the increased reliance on temporary contracts as a result of recent labor market reforms in European countries (Castellacci and Viñas-Bardolet, 2021). This trend has a significant impact on roles such as postdoctoral positions, which are characterized by employment uncertainty, limited career prospects and high workloads (Guidetti et al., 2022). As a result, these uncertainties discourage many talented young researchers from pursuing an academic career (Langenberg, 2001; Dorenkamp and Weiß, 2018), which has multiple implications for academia.

This study aims to deepen the understanding of job insecurity among research fellows and, in particular, to explore its impact on well-being. It seeks to identify distinct groups of early career researchers based on their experienced levels of job insecurity and its correlates - work engagement, emotional exhaustion and cynicism - essential dimensions of early career academic researcher well-being.

## 2 Job insecurity

In the constantly evolving world of the modern workplace, the nature and meaning of job insecurity has changed. Greenhalgh and Rosenblatt (1984) initially defined job insecurity as “the perceived powerlessness to maintain desired continuity in a threatened job situation” (p. 438), emphasizing the subjective aspect within the immediate work environment.

As defined by Heaney et al. (1994), Davy et al. (1997), Cheng and Chan (2008), and Shoss (2017), job insecurity reflects an individual perception of job continuity and includes concerns about job existence and job stability. Beyond the fear of immediate job loss, it includes concerns about valued aspects of work and career uncertainty (Greenhalgh and Rosenblatt, 1984; Hartley et al., 1991).

Although increasingly difficult to define and measure, it is important to note that job insecurity does not always signal imminent job loss (Sverke et al., 2002). Rather, it signifies the anticipation of a potentially stressful event when individuals perceive their employment to be at risk.

Research suggests that job insecurity has a profound effect on employees’ health, well-being, attitudes towards work, job perceptions and behaviors (De Witte, 1999). As engagement declines, individuals may distance themselves from the stressor by using coping mechanisms related to job insecurity (Cheng and Chan, 2008). When job insecurity arises, employees may adopt withdrawal strategies in response to decreased commitment. However, the relationship between job insecurity and employee commitment remains a subject of ongoing debate, with some studies showing no significant relationship (Sverke et al., 2002; Loi et al., 2011).

Despite extensive research on individual differences as moderators, there is still a gap in understanding how work environments influence the impact of job insecurity on employees’ work-related behaviors, as highlighted by Rosen et al. (2010). A more detailed identification of the effects of job insecurity on job performance is essential for the design of management interventions aimed at mitigating its negative effects.

Whereas there are many studies that have examined the relationship between different aspects of job insecurity and job outcomes using a variable-centered approach (De Witte et al., 2016), few studies have used a person-centered approach. The latter approach is advantageous because might help to identify high-risk subgroups and to shed light on the specific mechanisms by which variables (i.e., job insecurity and work-related health outcomes) differently work and combine each other across subgroups. In addition to a comprehensive measure, Wang et al. (2015) examined the specific effects of different dimensions of job insecurity. Currently, there is a lack of research on how work environments specifically influence the impact of job insecurity on employees’ work engagement. Further research is needed to identify the work conditions in which job insecurity has a significant impact on employees’ work engagement.

### 2.1 The situation of research fellows in Italy

In Italy, the path after obtaining a PhD degree is rather long and tortuous, and the reforms on public spending have led to significant cuts in research in public universities (Donina and Hasafendic, 2019).

After obtaining a PhD degree, according to the Law 240/2010, there are several steps in the academic career in the Italian public university system: (1) non-tenure-track postdoctoral researcher, (2) non-tenure-track type A researcher (three-year contract renewable for another 2 years after a positive evaluation by the university, at the end of which it is possible to access the type B position), (3) tenure-track type B researcher (three-year non-renewable contract, at the end of which direct access to the position of associate professor is possible, if the national scientific qualification is held and subject to a positive evaluation by the university), (4) associate professor and (5) full professor (Guidetti et al., 2022).

The Italian higher education system has experienced a decrease in public funding, resulting in the majority of scholarships being sourced from external funds. In both scenarios, doctoral students are actively involved in full-time research activities, typically working under the guidance of a faculty member who often serves as the principal investigator for the project. According to legal provisions, doctoral students can be granted research fellowships for a maximum duration of 6 years, whether consecutively or with gaps, with each fellowship spanning one to 3 years. Consequently, this system elevates the probability of interruptions between fellowships, which are inadequately compensated for through social protection measures.

This situation places the Italian system among the most precarious in Europe, and many researchers express the belief that their working conditions also impede their performance (Toscano et al., 2014).

If we refer to the population of early career researchers in STEM (science, technology, engineering, mathematics), their job satisfaction is mainly related to job insecurity (Grinstein and Treister, 2018), lack of work-life balance (Bogle et al., 2018), excessive workload (Dorenkamp and Weiß, 2018) and lack of organizational support (Miller and Feldman, 2015), which leads them to abandon their academic careers in favor of careers in other organizational settings. Some American studies have shown that young researchers at the beginning of their careers often developed poor well-being due to job insecurity and the long and often frustrating academic journey, which damaged their job satisfaction (Grinstein and Treister, 2018).

### 2.2 Work engagement, emotional exhaustion and cynicism in research fellows

For the purposes of this study, it was important to us to have a look at two of the key components of burnout: emotional exhaustion and cynicism. Emotional exhaustion refers to a chronic state of physical and emotional depletion, also described as feelings of extreme fatigue; cynicism describes an attitude characterized by detachment and hostility towards one’s work (Maslach and Jackson, 1981). In recent years, great importance has also been given to the dimension considered “opposite’ to burnout: work engagement. Work engagement is a multidimensional construct defined as a positive, fulfilling, work-related state of mind that is characterised by vigor, dedication, and absorption (González-Romá et al., 2006). Postdoctoral researchers who feel very capable when working on their research projects are generally highly motivated by the project and are also likely to show resilience when faced with challenges and difficulties. It has been suggested that dedication is the main form of commitment among early career researchers in the behavioral sciences (Vekkaila et al., 2012) which is shaped by various factors, encompassing a feeling of ownership, a sense of self-efficacy, and a sense of being part of a collaborative research team.

Disengagement, conversely, denotes a state of passivity or a lack of engagement in a specific task or activity, as highlighted by Fredricks et al. (2004) and Reeve et al. (2004). This state is often a consequence of prolonged work-related stress, as described by Lazarus (1998). Experiences of disengagement typically encompass negative emotions, decreased commitment, and the adoption of cynical attitudes toward one’s work in a broader context, as exemplified by Schaufeli et al. (2002). They are marked by feelings of ineffectiveness, psychological distress, and cynicism (Schaufeli et al., 2002). Professional ineffectiveness entails a reduced sense of accomplishment and a perception of one’s work as excessively demanding, psychological distress, on the other hand, manifests as anxiety, low energy, exhaustion, and heightened tension. The element of cynicism introduces a critical dimension as involve a waning and loss of interest in one’s work and the sense that its intrinsic purpose has been eroded, often leading to withdrawal from professional engagement due to diminished enthusiasm (Maslach and Leiter, 2008). Ultimately, the intricate facets of disengagement portray a vivid picture of the toll prolonged work-related stress has on the individual. It goes far beyond mere indifference, encompassing psychological strain, diminishing professional effectiveness, and a palpable loss of purpose in the workplace.

In the present study, we are interested in finding out whether there are homogeneous groups within our sample of researchers and how they are distributed in terms of job insecurity, work engagement, emotional exhaustion, and cynicism. We also analysed how the different clusters differ based on sociodemographic data.

## 3 Procedure

We conducted a survey in January 2022 among research fellows who had a position at a North Italian university in the fields of Science, Technology, Engineering and Mathematics (STEM). For the purpose of our survey, a research fellow is a PhD or graduate student with a scientific and professional curriculum vitae suitable for carrying out research activities, who is remunerated by means of ‘research grants’. Participants volunteered for the study without compensation, gave informed consent, and agreed to complete the questionnaire anonymously. The research adhered to the tenets of the Declaration of Helsinki of 1995 and its subsequent revisions, and all necessary ethical guidelines and legal requirements for human research in each country were strictly adhered to.

Of the 513 questionnaires sent out, 218 (42.7%) were returned correctly. A total of 137 respondents were males (62.6%) and 82 were females (37.4%), with mean age of 31.7 (SD = 5.08) and 78.1% (*N* = 171) of the participants were aged between 27 and 36 years. The gender distribution of the study sample reflects the gender distribution of STEM’s researcher population. This sample had a current contract of 1.5 years (SD = 0.9) but they had a history of collaboration with the institution of 4.09 years (SD = 3.44).

The *Individual Job Insecurity (IJI)* (*α* = 0.728; M = 9.71; SD = 3.39) was measured with six-item scale proposed by Låstad et al. (2015) (‘I feel insecure about the future of my job’).

*Cynicism* (CYN) (*α* = 0.908; M = 10.8; SD = 6.7) was measured using the five-item subscale of the Maslach Burnout Inventory-General Survey (It. version: Loera et al., 2014) (‘I have become less enthusiastic about my work’).

*Work Engagement* (WE) (*α* = 0.875; M = 39.08; SD = 9.52) was measured with the nine-item scale of Utrecht Work Engagement Scale (U-WES9) (Balducci et al., 2010) (At my work, I feel bursting with energy’).

*Emotional Exhaustion* (EE) (*α* = 0.865; M = 14.4; SD = 6.9) was measured using five-item scale of the Maslach Burnout Inventory-General Survey (Loera et al., 2014) (‘I feel used up at the end of the work day’).

Responses to the cynicism and dedication measures were provided on a scale ranging from 0 (‘Never’) to 6 (‘Every day’), while those on-the-job insecurity scale were rated on a four-point response scale ranging from 0 (‘Not at all’) to 3 (‘Completely’).

### 3.1 Group analysis

To understand if there are “hidden” subgroups of researchers in the sample based on the levels of precariousness and the relative consequences in terms of well-being, a latent profile analysis (Oberski, 2016) was conducted using the mixture model specification embedded in the statistical software Mplus. The scores of IJI, EE, CYN and WE were used as criterion variables to identify the latent classes. To determine the number of classes the approximate fit indices (Akaike Information Criterion-AIC, Bayesian Information Criterion-BIC and it adjusted version) were used, where lower values indicate superior model fitIn addition, we considered the Vuong-Lo–Mendell–Rubin adjusted likelihood ratio test (VLMR-LRT) which assess whether adding a class leads to a statistically significant improvement in model fit: a non-significant *p*-value for a k class solution lends support for the k - 1 class solution (Vermunt, 2024). Finally, the entropy coefficient and the average latent posterior probabilities predicting class membership for individuals were used to evaluate how accurately the selected model defines classes: an entropy value close to 1 is ideal and above 0.8 is sufficient, while probabilities between 0.80 and 0.90 are considered acceptable.

We estimated three nested models predicting the existence of 4, 3 or at least 2 latent classes (Table 1).

**Table 1:** LCA model fit and description of the selected 3 class solution.

Latent classes (K)	AIC	BIC	Adj. BIC	LRT test (K vs. K-1)	*p*	Entropy
2	5477.535	5521.533	5480.337	187.521	0.000	0.846
3	5435.594	5496.515	5439.474	50.081	0.004	0.766
4	5420.991	5498.835	5425.950	23.721	0.136	0.753
		Classification Probabilities for the Most Likely Latent Class Membership				
		1	2	3	% male	Mean age (SD)
1: “Safe & sound”		0.826	0.174	0.000	59.68	32.05 (6.15)
2: “Safe and not so sound”		0.077	0.896	0.027	64.22	31.23 (4.26)
3: “Neither safe or sound”		0.000	0.059	0.941	63.83	31.98 (4.79)

^*^Lo–Mendell–Rubin Adjusted LRT test K vs K-1 latent classes.

**Figure 1:**
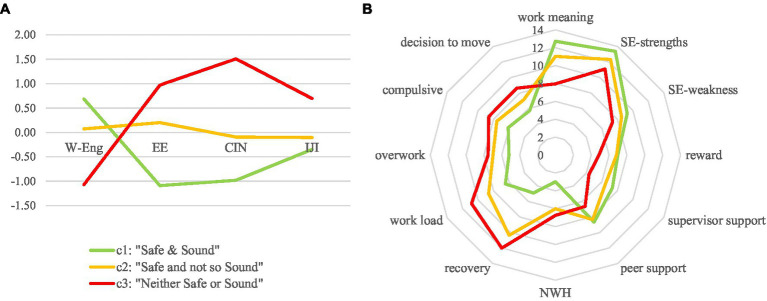
Displays the results of the cluster analysis. **(A)** Latent class profiles on standardized criterion variables and **(B)** nomological network of latest classes.

Considering all the diagnostics, we opted for the 3 classes solution (LRT tests significant, entropy above 0.8 and class membership over 0.9 or comprised between 0.8 and 0.9), that resulted a well- structured and meaningful partition of the examined researchers (Figure 1A). Moreover, the partition demonstrated an appreciable heuristic value since the three classes, or groups, accounted the variability of different convergent and divergent construct indicators (Figure 1B), denoting a valid nomological network.

Table 1 shown LCA model fit and description of the selected 3 class solution.

The cluster analysis assigns a total of 218 scholars into three classes. The first cluster, named ‘Safe & Sound’, comprises 28.4% of the scholars, the second cluster, named ‘Safe not so Sound’, comprises 50.0%, and the third cluster, named ‘Neither Safe nor Sound’, comprises 19.4%. The labels for the clusters were assigned based on the average responses. The first group, in which scholars are more engaged, shows lower levels of emotional exhaustion and cynicism, which are also associated with lower levels of individual job insecurity. The scholars in the second group appear to be more emotionally exhausted and detached from their work. This group has low levels of work engagement and greater individual job insecurity. The third group has the lowest levels of work engagement and the highest levels of emotional exhaustion and detachment from work. These are also associated with higher levels of job insecurity.

As previously reported, the ‘Safe & Sound’ group exhibited higher levels of work engagement (*M* = 45.52) than the other two groups (Safe not so sound, *M* = 39.9; ‘Neither Safe or Sound’, *M* = 29.5). Consistent with this finding, the third group reported higher levels of emotional exhaustion (*M* = 21.2) than the other two groups (‘Safe & Sound’, *M* = 6.9; ‘Safe and not so Sound’, *M* = 15.9), as well as hostility towards their work (‘Safe & Sound’, *M* = 4.2; Safe and not so Sound, *M* = 10.2; ‘Neither Safe or Sound’, *M* = 21.06).

These results are also associated with levels of job insecurity. In the first cluster, where there are higher levels of engagement, job insecurity is lower (*M* = 8.5) than in the other two clusters where job insecurity increases (Safe not so Sound, *M* = 9.37; ‘Neither Safe or Sound’, *M* = 12.09) as work engagement decreases.

The validity of the partition was also tested with respect to other criterion variables that are considered antecedents of well-being. These variables include workaholism, which consists of overwork (*F* = 12.4) and compulsive work (*F* = 10.4); support from colleagues (*F* = 7.6) and supervisors (*F* = 19.9); and self-efficacy, regarding weaknesses (*F* = 14.1) and strengths (*F* = 15.6). All F-tests, controlled by Manova, were statistically significant (*p* < 0.05).

Jansen et al. (2002) stated that the need for recovery is a precursor to prolonged fatigue or psychological distress (p. 324). In everyday situations, individuals express their need for recovery as a desire to recharge their batteries. After a busy day at work, individuals may have reached a high level of activity, implying a high level of arousal, and consequently, they may have exhausted many of their resources. In such situations, individuals may require more time to recover, and the need for recovery may persist beyond the immediate rest period. Inadequate recovery may result in individuals starting the next working day with a high need for recovery. Like the previous variables, the *F*-test is statistically significant (*F* = 37.6).

Equally important in the area of occupational well-being is the support of colleagues and supervisors (Schreurs et al., 2012). Furthermore, according to theoretical and empirical evidence, supervisor support has been primarily viewed as a buffer for the link between individual job insecurity and well-being outcomes (Schreurs et al., 2012; Cheng et al., 2014). The sample demonstrates significantly higher levels of supervisor support within the type 1 cluster (‘Safe & Sound’). Additionally, social support from co-workers was found to be associated with a positive mood and higher commitment, which is in agreement with the literature. The type 1 cluster (‘Safe & Sound’) also shows higher levels of peer support and work commitment.

Self-efficacy is widely recognized as a crucial resource for coping with the unpredictability of today’s work and career environment (Hirschi et al., 2013; Garcia et al., 2015). Self-efficacy refers to an individual’s confidence in their ability to successfully pursue goals and cope with difficulties that may arise in the process. In cluster 1, self-efficacy is higher than in the other two clusters, both in terms of strengths and weaknesses.

## 4 Discussion

Compared to the data in the literature, our study shows that research fellows at a university in the STEM disciplines do not exhibit significant levels of individual job insecurity. This finding may be due to the motivation of these researchers, who choose to pursue this path despite many job offers outside the university context.

It is interesting to note the gender distribution, where women, although in the minority, are more representative within cluster 1 ‘Safe & Sound’. This observation contrasts with traditional gender stereotypes in academic settings, suggesting that women in STEM fields may possess distinctive characteristics contributing to higher commitment levels (van Veelen and Derks, 2022; Schmader, 2023).

In line with the literature, support from supervisors and peers is greater in the committed type, showing its importance in reducing job insecurity.

It is reasonable to speculate that individuals who believe in their ability to secure a new job might not respond as negatively to perceptions of job insecurity compared to those who lack such confidence. The relationship between job insecurity and its outcomes has not been extensively explored, particularly in terms of how employment dynamics moderate these relationships.

From a practical point of view, findings from the present study suggest that job insecurity represents a detrimental condition for employees as it always appears in combination with high level of burnout and, in the case of the “neither safe or sound” subgroup, also in presence of poor work engagement. In this view, in order to reduce stress and support motivation, national reforms that increase the opportunity for post-doc researchers to reach tenure-track positions would be beneficial. When this is not feasible, due to lack of financial resources, academic organizations should pay great attention to the improvement of the quality of the psychosocial work environment. In particular, it was found that the presence of a supportive social environment (both from superior and peers) might mitigate the likelihood for the individual to fall in the subgroup exposed at the highest risk for psychosocial well-being (“neither safe or sound”). Accordingly, implementing programmes addressed at empowering leadership skills among research group coordinators might help them to develop awareness and capability in holding their role of mentoring (and not only merely of coordination) towards younger and precarious employees. In addition, organization should offer both formal and informal occasions of socialization among peers; some example are: organizing meetings of peer confrontation regarding research projects and academic life; arranging areas in the workplace for socializing, dining and relaxing specifically dedicated to PhD and post-doc researchers. These initiatives might favor sharing experience among precarious employees and strengthen their feeling of being part of a supportive social network, thus enhancing job engagement and reducing stress.

### 4.1 Strengths, limitations and future research

The study offered some insight regarding the role of employment dynamics in the relationship between job insecurity and its outcomes opens up new research avenues. The assumption that individuals who are confident in their ability to secure alternative employment may respond differently to perceptions of job insecurity warrants a more detailed investigation. This suggests the need for longitudinal studies to understand how employment dynamics influence the psychological responses to job insecurity over time, providing a more comprehensive understanding of the complex interplay between individual beliefs, employment opportunities, and the experience of job insecurity.

The long-term effects of job insecurity remain unclear, so longitudinal research is needed to understand their evolution. A nuanced relationship exists between job insecurity and outcomes, with studies suggesting that initial levels of job insecurity predict negative outcomes in the future (Garst et al., 2000). The study cross-sectional design gives us a snapshot of job insecurity among researchers, but it limits our ability to draw causal relationships. Future research could explore job insecurity evolution and long-term effects using longitudinal methods. Research fellows in a specific region that is specific to Italy, which limited its generalizability. The use of self-reported data introduces the possibility of response bias. A more diverse sample across different geographical locations could provide a broader perspective. Understanding how these experiences unfold may provide valuable insights for intervention strategies. Adding qualitative insights to quantitative data can provide a more comprehensive understanding of the contextual factors influencing job insecurity. Focus groups and in-depth interviews can help capture subtleties that quantitative measures cannot. A critical component of mitigating job insecurity’s negative effects is the effectiveness of various interventions. Future studies may explore organizational factors that may exacerbate or alleviate job insecurity, including mentoring programs, training initiatives, and organizational support structures. Researchers can contribute to a more nuanced understanding of job insecurity in STEM fields and develop targeted interventions to support the well-being of research fellows by addressing these limitations and pursuing these future research directions.

## Data availability statement

The raw data supporting the conclusions of this article will be made available by the authors, without undue reservation.

## Ethics statement

Ethical approval was not required for the studies involving humans because The research conformed to the provisions of the Declaration of Helsinki in 1995 (and subsequent revisions), and all ethical guidelines were followed as required for conducting human research, including adherence to the legal requirements of Italy. Additional ethical approval was not required since there was no treatment including medical, invasive diagnostics or procedures causing participants psychological or social discomfort, nor were patients the subject of data collection. The studies were conducted in accordance with the local legislation and institutional requirements. The participants provided their written informed consent to participate in this study.

## Author contributions

GB: Formal analysis, Methodology, Writing – original draft, Writing – review & editing. SV: Writing – original draft, Writing – review & editing. LB: Writing – original draft, Writing – review & editing. DC: Conceptualization, Supervision, Writing – original draft, Writing – review & editing. BL: Conceptualization, Formal analysis, Methodology, Supervision, Writing – original draft, Writing – review & editing.

## References

[ref1] Armstrong-Stassen M. (1993). Production workers' reactions to a plant closing: the role of transfer, stress, and support. Anxiety Stress Coping 6, 201–214. doi: 10.1080/10615809308248380

[ref2] Arnold H. J. Feldman D. C. (1982). A multivariate analysis of the determinants ofjob turnover. J. Appl. Psychol. 67, 350–360. doi: 10.1037/0021-9010.67.3.350

[ref3] Ashford S. J. Lee C. Bobko P. (1989). Content, cause, and consequences of job insecurity: a theory-based measure and substantive test. Acad. Manag. J. 32, 803–829. doi: 10.2307/256569

[ref4] Balducci C. Fraccaroli F. Schaufeli W. B. (2010). Psychometric properties of the Italian version of the Utrecht work engagement scale (UWES-9): a cross-cultural analysis. Eur. J. Psychol. Assess. 26, 143–149. doi: 10.1027/1015-5759/a000020

[ref7] Bogle D. Compton-Daw E. Elvidge L. Gavaghan D. Henstock J. Jones D.. (2018). Review of the concordat to support the career development of researchers. UK: Concordat Strategy Group.

[ref8] Brockner J. (1988). “The effects of work layoffs on survivors: research, theory, and practice” in Research in organisational behavior. eds. Staw B. M. Cummings L. L. (Greenwich, CT: JAI Press).

[ref9] Castellacci F. Viñas-Bardolet C. (2021). Permanent contracts and job satisfaction in academia: evidence from European countries. Stud. High. Educ. 46, 1866–1880. doi: 10.1080/03075079.2019.1711041

[ref10] Cheng G. H.-L. Chan D. K.-S. (2008). Who suffers more from job insecurity? A meta-analytic review. Appl. Psychol. Int. Rev. 57, 272–303. doi: 10.1111/j.1464-0597.2007.00312.x

[ref11] Cheng T. Mauno S. Lee C. (2014). Do job control, support, and optimism help job insecure employees? A three-wave study of buffering effects on job satisfaction, vigor and work-family enrichment. Soc. Indic. Res. 118, 1269–1291. doi: 10.1007/s11205-013-0467-8

[ref12] Davy J. A. Kinicki A. J. Scheck C. L. (1997). A test of job security's direct and mediated effects on withdrawal cognitions. J. Organ. Behav. 18, 323–349. doi: 10.1002/(SICI)1099-1379(199707)18:4<323::AID-JOB801>3.0.CO;2-#

[ref13] De Witte H. (1999). Job insecurity and psychological well-being: review of the literature and explo-ration of some unresolved issues. Eur. J. Work Organ. Psy. 8, 155–177. doi: 10.1080/135943299398302

[ref14] De Witte H. Pienaar J. De Cuyper N. (2016). Review of 30 years of longitudinal studies on the association between job insecurity and health and well-being: is there causal evidence? Aust. Psychol. 51, 18–31. doi: 10.1111/ap.12176

[ref15] Donina D. Hasafendic S. (2019). Higher education institutional governance reforms in the Netherlands, Portugal and Italy: a policy translation perspective addressing the homogeneous/heterogeneous dilemma. High. Educ. Q. 73, 29–44. doi: 10.1111/hequ.12183

[ref16] Dorenkamp I. Weiß E. E. (2018). What makes them leave? A path model of postdocs’ intentions to leave academia. High. Educ. 75, 747–767. doi: 10.1007/s10734-017-0164-7

[ref17] Fredricks J. A. Blumenfeld P. C. Paris A. H. (2004). School engagement: potential of the concept, state of the evidence. Rev. Educ. Res. 74, 59–109. doi: 10.3102/00346543074001059

[ref18] Gallie D. Felstead A. Green F. Inanc H. (2017). The hidden face of job insecurity. Work Employ. Soc. 31, 36–53. doi: 10.1177/0950017015624399

[ref19] Garcia P. R. J. M. Restubog S. L. D. Bordia P. Bordia S. Roxas R. E. O. (2015). Career optimism: the roles of contextual support and career decision-making self-efficacy. J. Vocat. Behav. 88, 10–18. doi: 10.1016/j.jvb.2015.02.004

[ref9002] Garst H. Frese M. Molenaar P. (2000). The temporal factor of change in stressor–strain relationships: a growth curve model on a longitudinal study in East Germany. J. Appl. Psychol. 85, 417. 10900816 10.1037/0021-9010.85.3.417

[ref20] González-Romá V. Schaufeli W. B. Bakker A. B. Lloret S. (2006). Burnout and work engagement: independent factors or opposite poles? J. Vocat. Behav. 68, 165–174. doi: 10.1016/j.jvb.2005.01.003

[ref21] Goulden M. Mason M. A. Frasch K. (2011). Keeping women in the science pipeline. Ann. Am. Acad. Polit. Soc. Sci. 638, 141–162. doi: 10.1177/0002716211416925

[ref23] Greenhalgh L. Rosenblatt Z. (1984). Job insecurity: toward conceptual clarity. Acad. Manag. Rev. 9, 438–448. doi: 10.2307/258284

[ref24] Grinstein A. Treister R. (2018). The unhappy postdoc: a survey basedstudy. F1000Research 6:1642. doi: 10.12688/f1000research.12538.2 PMC5958315 29946421

[ref25] Guidetti G. Converso D. Di Fiore T. Viotti S. (2022). Cynicism and dedication to work in post-docs: relationships between individual job insecurity, job insecurity climate, and supervisor support. Eur. J. High. Educ. 12, 134–152. doi: 10.1080/21568235.2021.1900743

[ref26] Hartley J. Jacobson D. Klandermans B. van Vuuren T. (1991). Job insecurity: Coping with jobs at risk. London: Sage.

[ref9004] Heaney C. A. Israel B. A. House J. S. (1994). Chronic job insecurity among automobile workers: effects on job satisfaction and health. Soc. Sci. Med. 38, 1431–1437. 8023192 10.1016/0277-9536(94)90281-x

[ref27] Hirschi A. Lee B. Porfeli E. J. Vondracek F. W. (2013). Proactive motivation and engagement in career behaviors: investigating direct, mediated, and moderated effects. J. Vocat. Behav. 83, 31–40. doi: 10.1016/j.jvb.2013.02.003

[ref28] Jansen N. W. H. Kant I. van den Brandt P. A. (2002). Need for recovery in the working population: description and associations with fatigue and psychological distress. Int. J. Behav. Med. 9, 322–340. doi: 10.1207/S15327558IJBM0904_03, PMID: 12512472

[ref29] Keim A. C. Landis R. S. Pierce C. A. Earnest D. R. (2014). Why do employees worry about their jobs? A meta-analytic review of predictors of job insecurity. J. Occup. Health Psychol. 19, 269–290. doi: 10.1037/a0036743, PMID: 24796228

[ref30] Langenberg H. (2001). Uncertainty of short-term contracts is turning talent away from science. Nature 410, 849–850. doi: 10.1038/35071226

[ref31] Låstad L. Berntson E. Näswall K. Lindfors P. Sverke M. (2015). Measuring quantitative and qualitative aspects of the job insecurity climate: scale validation. Career Dev. Int. 20, 202–217. doi: 10.1108/CDI-03-2014-0047

[ref32] Lazarus R. S. (1998). “The stress and coping paradigm” in Fifty years of the research and theory of RS Lazarus: An analysis of historical and perennial Issues, 182–220. Psychology Press.

[ref33] Loera B. Converso D. Viotti S. (2014). Evaluating the psychometric properties of the Maslach burnout inventory-human services survey (MBI-HSS) among Italian nurses: how many factors must a researcher consider? PLoS One 9:e114987. doi: 10.1371/journal.pone.0114987, PMID: 25501716 PMC4264862

[ref34] Loi R. Ngo H. Y. Zhang L. Q. Lau V. P. (2011). The interaction between leader-member exchange and perceived job security in pre-dicting employee altruism and work performance. J. Occup. Organ. Psychol. 84, 669–685. doi: 10.1348/096317910X510468

[ref35] Maslach C. Jackson S. E. (1981). The measurement of experienced burnout. J. Organ. Behav. 2, 99–113. doi: 10.1002/job.4030020205

[ref36] Maslach C. Leiter M. P. (2008). Early predictors of job burnout and engagement. J. Appl. Psychol. 93, 498–512. doi: 10.1037/0021-9010.93.3.498, PMID: 18457483

[ref38] Miller J. M. Feldman M. P. (2015). Isolated in the lab: examining dissatisfaction with postdoctoral appointments. J. High. Educ. 86, 697–724. doi: 10.1080/00221546.2015.11777380

[ref39] Oberski D. (2016). “Mixture models: latent profile and latent class analysis” in Modern statistical methods for HCI. Human–computer interaction series. eds. Robertson J. Kaptein M. (Cham: Springer). doi: 10.1007/978-3-319-26633-6_12

[ref40] Reeve J. Jang H. Carrell D. Jeon S. Barch J. (2004). Enhancing students' engagement by increasing teachers' autonomy support. Motiv. Emot. 28, 147–169. doi: 10.1023/B:MOEM.0000032312.95499.6f

[ref42] Rosenblatt Z. Ruvio A. (1996). A test of a multidimensional model of job insecurity: the case of Israeli teachers. J. Organ. Behav. 17, 587–605. doi: 10.1002/(SICI)1099-1379(199612)17:1+<587::AID-JOB825>3.0.CO;2-S

[ref9003] Rosen C. C. Chang C. H. Djurdjevic E. Eatough E. (2010). Occupational stressors and job performance: an updated review and recommendations. New developments in theoretical and conceptual approaches to job stress, 1–60. Bingley, UK: Emerald.

[ref43] Schaufeli W. B. Martinez I. M. Pinto A. M. Salanova M. Bakker A. B. (2002). Burnout and engagement in university students: a cross-national study. J. Cross-Cult. Psychol. 33, 464–481. doi: 10.1177/0022022102033005003

[ref45] Schmader T. (2023). Gender inclusion and fit in STEM. Annu. Rev. Psychol. 74, 219–243. doi: 10.1146/annurev-psych-032720-043052 35961037

[ref46] Schreurs B. H. Hetty van Emmerik I. J. Günter H. Germeys F. (2012). A weekly diary study on the buffering role of social support in the relationship between job insecurity and employee performance. Hum. Resour. Manag. 51, 259–279. doi: 10.1002/hrm.21465

[ref48] Shoss M. K. (2017). Job insecurity: an integrative review and agenda for future research. J. Manag. 43, 1911–1939. doi: 10.1177/0149206317691574

[ref9005] Sverke M. Hellgren J. (2001). Exit, voice and loyalty reactions to job insecurity in Sweden: Do unionized and non‐unionized employees differ? Br. J. Ind. Relat. 39, 167–182.

[ref49] Sverke M. Hellgren J. Näswall K. (2002). No security: a Meta-analysis and review of job insecurity and its consequences. J. Occup. Health Psychol. 7, 242–264. doi: 10.1037/1076-8998.7.3.242 12148956

[ref50] The jamovi project (2022). jamovi. (Version 2.3) [Computer Software]. Available at: https://www.jamovi.org

[ref51] Toscano E. Coin F. Giancola O.. (2014). RICERCARSI – Indagine sui percorsi di vita e lavoro del precariato universitario. Available at: http://www.roars.it/online/ricercarsi-indagine-sui-percorsi-di-vita-e-lavoro-nel-precariato-universitario

[ref52] van Veelen R. Derks B. (2022). Equal representation does not mean equal opportunity: women academics perceive a thicker glass ceiling in social and behavioral fields than in the natural sciences and economics. Front. Psychol. 13:790211. doi: 10.3389/fpsyg.2022.790211, PMID: 35369222 PMC8966382

[ref53] Vander Elst T. Näswall K. Bernhard-Oettel C. De Witte H. Sverke M. (2016). The effect of job insecurity on employee health complaints: a within-person analysis of the explanatory role of threats to the manifest and latent benefits of work. J. Occup. Health Psychol. 21, 65–76. doi: 10.1037/a0039140, PMID: 25894197

[ref54] Vekkaila J. Pyhältö K. Hakkarainen K. Keskinen J. Lonka K. (2012). Doctoral students’ key learning experiences in the natural sciences. Int. J. Res. Dev. 3, 154–183. doi: 10.1108/17597511311316991

[ref55] Vermunt J. K. (2024). The Vuong-Lo-Mendell-Rubin test for latent class and latent profile analysis: a note on the different implementations in Mplus and latent GOLD. Methodology 20, 72–83. doi: 10.5964/meth.12467

[ref56] Wang H. J. Lu C. Q. Siu O. L. (2015). Job insecurity and job performance: the moderating role of organizational justice and the mediating role of work engagement. J. Appl. Psychol. 100, 1249–1258. doi: 10.1037/a0038330, PMID: 25402953

